# Supplementary material for: Ternary structure of the outer membrane transporter FoxA with resolved signalling domain provides insights into TonB-mediated siderophore uptake
Source: eLife. 2019 Aug 6;8:e48528. doi: 10.7554/eLife.48528 (PMC6699858; doi:10.7554/eLife.48528)
Supplement: Supplementary file 1. [file elife-48528-supp1.docx]

**Supplementary Information for:**

**Ternary structure of the outer membrane transporter FoxA with resolved signaling domain provides insights into TonB-mediated siderophore uptake**

Inokentijs Josts^1^*, Katharina Veith^1^ and Henning Tidow^1^*

^1^ The Hamburg Centre for Ultrafast Imaging & Department of Chemistry, Institute for Biochemistry and Molecular Biology, University of Hamburg, Martin-Luther-King-Platz 6, 20146 Hamburg, Germany

* Corresponding authors:

Inokentijs Josts

University of Hamburg, Department of Chemistry, Institute for Biochemistry and Molecular Biology, Martin-Luther-King-Platz 6, D-20146 Hamburg, Germany

Tel: +49 40428389047

e-mail: josts@chemie.uni-hamburg.de

Henning Tidow

University of Hamburg, Department of Chemistry, Institute for Biochemistry and Molecular Biology, Martin-Luther-King-Platz 6, D-20146 Hamburg, Germany

Tel: +49 40428388984

e-mail: tidow@chemie.uni-hamburg.de

**Supplementary tables**

**Table 1:** **Data collection and refinement statistics**

|  | Apo FoxA  (pdb: 6I98) | FoxA-ferrioxamine B (pdb: 6I96) | FoxA-ferrioxamine B-TonB_Ct_ complex  (pdb: 6I97) |
| --- | --- | --- | --- |
| **Data collection** |  |  |  |
| **Beamline** | PETRA III, P13 | BESSY 14.1 | PETRA III, P13 |
| Space group | P6_3_22 | P3_2_21 | P2_1_2_1_2_1_ |
| Cell dimensions |  |  |  |
| *a*, *b*, *c* (Å) | 174.6, 174.6, 180.2 | 94.9, 94.9, 177.6 | 163.6, 174.4, 214.1 |
| α, β, γ (°) | 90, 90, 120 | 90, 90, 120 | 90, 90, 90 |
| Resolution (Å) | 48.53-2.80 (2.91-2.80) | 82.18-1.85 (1.88-1.85) | 48.76-3.35 (3.41-3.35) |
| *R*_merge_ | 0.206 (1.56) | 0.15 (1.29) | 0.286 (1.8) |
| *R_meas_* | 0.226 (1.72) | 0.16 (1.45) | 0.34 (2.14) |
| *I* / σ*I* | 10 (1.6) | 11.2 (1.0) | 5.6 (1.0) |
| *CC_1/2_* | 0.99 (0.55) | 0.99 (0.28) | 0.99 (0.389) |
| Completeness (%) | 100 (99.9) | 99.2 (92.6) | 100 (100) |
| Redundancy | 10.7 (10.6) | 17 (4.7) | 6.7 (6.5) |
|  |  |  |  |
| **Refinement** |  |  |  |
| Resolution (Å) | 2.8 | 1.85 | 3.35 |
| No. reflections | 38331 (2769) | 75099 (5174) | 84137 (6117) |
| *R*_work_ / *R*_free_ | 0.21/0.26 | 0.18/0.22 | 0.23/0.26 |
| No. atoms | 5527 | 5945 | 13437 |
| Protein | 5325 | 5325 | 13357 |
| Ligand/ion | 191 | 363 | 80 |
| Water | 14 | 257 |  |
| *B*-factors |  |  |  |
| Protein | 50.2 | 49.7 | 68.1 |
| Ligand/ion | 87.2 | 81.8 | 79.3 |
| Water | 43.4 | 47.8 |  |
| R.m.s. deviations |  |  |  |
| Bond lengths (Å) | 0.02 | 0.027 | 0.02 |
| Bond angles (°) | 2.22 | 3.18 | 2.52 |
|  |  |  |  |
|  |  |  |  |
|  |  |  |  |

*Values in parentheses are for highest-resolution shell.

**Table 2: Summary of all the thermodynamic parameters determined by ITC**

| **Interaction studied** | **K_d_** | **ΔH (kcal mol^-1^)** | **TΔS (kcal mol^-1^)** |
| --- | --- | --- | --- |
| FoxA with TonB_Ct_ | 111 ± 6.5 nM | -10.1 ± 0.8 | -0.69 ± 0.39 |
| FoxA-foaB with TonB_Ct_ | 6.6 ± 1.2 nM | -18.1 ± 0.9 | -6.62 ± 0.49 |
| FoxA with foaB | 210 ± 44 nM | -4.9 ± 0.6 | 4.2 ± 0.54 |
| FoxA-TonB_Ct_ with foaB | 110 ± 50 nM | -26.7 ± 0.6 | -16.8 ± 1.14 |
